# Thoracic delirium index for predicting postoperative delirium in elderly patients following thoracic surgery: A retrospective case‐control study

**DOI:** 10.1002/brb3.3379

**Published:** 2024-01-08

**Authors:** Jianli Li, Jing Liu, Mingming Zhang, Jing Wang, Meinv Liu, Dongdong Yu, Junfang Rong

**Affiliations:** ^1^ Department of Anesthesiology Hebei General Hospital Shijiazhuang City China; ^2^ Graduate Faculty Hebei North University Zhangjiakou City China

**Keywords:** delirium, elderly patients, postoperative period, risk factors, thoracic surgery

## Abstract

**Background:**

Postoperative delirium (POD) is an acute neurological complication in the elderly undergoing thoracic surgery and can result in serious adverse consequences.

**Aims:**

This study aimed to identify the related risk factors for POD following thoracic surgery, primarily focusing on preoperative serum biomarkers, and further to establish a novel delirium index to better predict POD.

**Methods:**

A total of 279 patients aged ≥60 years who underwent elective thoracic surgery from August 2021 to August 2022 were enrolled in this observational study. The platelet‐to‐white blood cell ratio (PWR) was calculated as number the of platelets divided by the number of white blood cells. POD was defined by the confusion assessment method twice daily during the postoperative first 3 days. Multivariate regression analysis was performed to identify all potential variables for POD. Moreover, a novel thoracic delirium index (TDI) was developed based on the related risk factors. The accuracy of TDI and its component factors in predicting POD was determined by the curve of receiver operating characteristic (ROC).

**Results:**

In total, 25 of 279 patients developed POD (8.96%). Age, PWR, and average pain scores within the first 3 days after surgery were regarded as the independent risk factors for POD. Moreover, the ROC analysis showed the TDI, including age, PWR, and average pain scores within the first 3 days after surgery, can more accurately predict POD with the largest area under the curve of 0.790 and the optimal cutoff value of 9.072, respectively.

**Conclusion:**

The TDI can scientifically and effectively predict POD to provide optimal clinical guidance for older patients after thoracic surgery.

## Graphical abstract

Postoperative delirium (POD) can be prevented in elderly patients undergoing thoracic surgery by identifying its risk factors. The thoracic delirium index could early identify people at risk of POD, thus improving the prognosis of patients. Clinicians can reduce POD in the elderly after thoracic surgery by improving nutritional status before surgery, monitoring infectious signs using multiple objective laboratory results, and managing the pain after surgery.

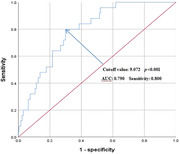

## 1 INTRODUCTION

The number of geriatric patients undergoing thoracic surgery is increasing with the aging of the population (Castillo, 2018). Thoracic surgery is a complex surgical procedure, which often leads to various postoperative complications, including delirium and pneumonia, etc. (Fernandez et al., 2018). The proportion of postoperative complications ranges from 12% to 47% in older patients following thoracic surgery in China, which has attracted extensive attention from clinicians (Han et al., 2019). Postoperative delirium (POD), a usual complication in senile patients after thoracic surgery, is identified as a reversible and comprehensive cognitive impairment, manifested as acute and transient changes in consciousness level, inattention, and disorientation after surgery (Oh & Park, 2019). POD usually occurs in the first 3 postoperative days and is considered as a harbinger of early postoperative cognitive decline (POCD) development (Glumac et al., 2019). It is reported that approximately 5–8% of patients will develop POD undergoing thoracic surgery, mainly in their 50s (Ozyurtkan et al., 2010; Yildizeli et al., 2005). Due to the decline in physiological function, older patients might have an increased risk of POD. POD is closely associated with various negative outcomes, namely longer hospitalization, increased economic costs, as well as increased mortality (Aung Thein et al., 2020; Gou et al., 2021). Therefore, it may be of great importance to explore appropriate methods for preventing and treating POD.

To date, the mechanism of POD remains not fully understood, and there are few related treatment schemes. Fortunately, it is generally accepted that prevention is the most effective strategy to manage POD (Janssen et al., 2019). Numerous studies indicated that POD is complex, depending on the interaction of multiple factors (Seiler et al., 2020; Zipser et al., 2021). Hence, early recognition of relevant risk factors may exert a significant effect on preventing POD. As previous studies mentioned, common risk factors for POD were mainly divided into three parts: preoperative factors (e.g., age and cerebrovascular diseases, etc.), intraoperative factors (e.g., operation time and blood transfusion, etc.), and postoperative factors (e.g., low hemoglobin, albumin, and sodium, etc.) (Baek et al., 2020; Visser et al., 2021; Zhu et al., 2020). In addition, the pathogenesis of POD and POCD is similar, in which neuroinflammation plays an important role. A study by Glumac et al. (2017) demonstrated that inflammatory response was involved in the development of POCD and preoperative administration of dexamethasone significantly decreased the risk of early POCD after cardiac surgery by reducing the level of some inflammatory factors, such as C‐reactive protein (CRP). Similarly, CRP and Interleukin‐6 (IL‐6) were also reported to be related to the occurrence of POD (Noah et al., 2021; Taylor et al., 2022). However, it is difficult, expensive, and inconvenient to measure these inflammatory mediators. Instead, the relationship between derivatives in leukocytes and POD has been extensively explored in recent years. Previous studies demonstrated that the neutrophil‐to‐lymphocyte ratio (NLR), platelet‐to‐lymphocyte ratio (PLR), as well as platelet‐to‐white blood cell ratio (PWR) have been acknowledged as potential predictors of POD in head and neck free‐flap reconstruction or abdominal surgery (Ida et al., 2020; Kinoshita et al., 2021). Also, a recent study showed that the monocyte‐to‐lymphocyte ratio (MLR) was independently correlated with POD in intensive care unit (ICU) patients after cardiac surgery (Su et al., 2022). Furthermore, the above inflammatory indexes were capable of predicting postoperative complications and long‐term prognosis in thoracic oncology surgery (Ginesu et al., 2022; Tsukioka et al., 2022). Nevertheless, the predictive role of these serum biomarkers in POD following thoracic surgery has not been investigated so far.

In this context, our study attempted to ascertain some leukocyte derivatives as strong predictors of POD in senile patients following thoracic surgery. Furthermore, we would establish a novel thoracic delirium index (TDI) based on the relevant variables, thus better assessing the risk of delirium and providing evidence support for clinical management to prevent POD.

## 2 METHODS

### 2.1 Study setting

This single‐center retrospective case‐control study was approved by the Medical Ethics Committee of Hebei General Hospital (2022117) and was registered at the Chinese Clinical Trial Registry (ChiCTR2200063556). Enrolled patients at Hebei General Hospital from August 2021 to August 2022 meet the following criteria: (1) patients aged ≥60 years old; (2) patients underwent elective thoracic surgery; (3) patients were operated under general anesthesia, and the postoperative hospital stay was no less than 3 days.

Patients who developed delirium before surgery were unable to communicate, did not wake up to enter into ICU postoperatively, and lacked complete data were excluded.

### 2.2 Perioperative management and data collection

All subjects were managed by the same surgical and anesthesia team, and the schemes of their anesthesia induction and maintenance were standardized. None of the included patients used epidural anesthesia in the perioperative period. Meanwhile, the same formulation of patient‐controlled intravenous analgesia was administered according to the needs of the patients and surgery.

Perioperative data were acquired from electronic medical records, consisting of demographic, clinical, as well as laboratory data. Demographic data contained sex, age, educational degree, American Society of Anesthesiologists grade, and body mass index. The clinical data were divided into three sections according to different time phases: preoperative, intraoperative, and postoperative.

Preoperative data included smoking history, alcohol abuse, anxiety and depression history, cognitive impairment, preoperative comorbidities, and usage of medications. The Charlson comorbidity Index (CCI) was adopted to evaluate preoperative comorbidities, with a weighted index by calculating scores for 19 different comorbidities (Charlson et al., 2022). A hospital anxiety and depression scale was used to assess anxiety or depression (Annunziata et al., 2020). Intraoperative data, such as types of diseases (lung cancer, esophageal tumor, tracheal tumor, hiatal hernia, mediastinal tumor, and chest wall tumor), surgical methods (open or endoscopic), opioid usage, operation time, estimated blood loss volume and blood infusion, etc., were obtained from the anesthesia record sheet. Some postoperative factors including average pain scores during the postoperative first 3 days and the postsurgical stay, etc., were also collected in this study. The visual analogue scale (VAS) was applied to evaluate postoperative pain (da Costa et al., 2021).

In addition, some preoperative laboratory indicators were extracted, namely hemoglobin, albumin, white blood cell count, and platelet, etc. The NLR, PLR, PWR, MLR, and GNRI were defined using the following equation: NLR (neutrophil to lymphocyte ratio); PLR (platelet to lymphocyte ratio); PWR (platelet to white blood cell ratio); MLR (monocyte to lymphocyte ratio); Geriatric nutritional risk index (GNRI) = 1.489 × serum albumin (g/L) + 41.7 × present weight/ideal weight (kg). The ideal body weight was derived from the Lorentz equation (Zhao et al., 2020).

All data were identified as potential variables for POD, which were obtained independently from medical records by two investigators.

### 2.3 Neurological assessment

The mental status was assessed the day before surgery using a mini‐mental state examination scale (MMSE). The MMSE score below 27 was identified as cognitive impairment (Tsang et al., 2019).

The confusion assessment method (CAM), a common diagnostic tool for POD, was performed to evaluate POD by trained anesthesiologists twice daily (08:00–10:00 am and 6:00–8:00 pm) within the postoperative first 3 days. Based on the *Diagnostic and Statistical Manual of Mental Disorders* criteria, CAM has been widely used by nonpsychiatric clinicians to screen high‐risk populations for POD with a high sensitivity of 94–100% and a specificity of 90–95%, including the following four aspects: (1) an acute fluctuation in consciousness level, (2) a transient change in mental condition, (3) inattention, and (4) confusion of the thought. Delirium was diagnosed as the appearance of (1) and (2), together with (3) or (4), both on any of the postoperative 3 days (Inouye et al., 1990).

### 2.4 Sample size

The statistical method in this study was multivariate logistic regression analysis, so we referred to the literature on the simulation of the number of variable events in logistic regression analysis by Peduzzi et al. (1996), which showed that at least 6–10 patients were needed for each independent variable event (EPV) to fully estimate the effect of the binary regression model. If 3 EPV were used to predict POD, the number of POD occurrences was at least 18 cases. Meanwhile, based on a study that noted the POD incidence (π) following thoracic surgery was about 8% (Ozyurtkan et al., 2010), and the total sample size was at least 225 cases according to the operational rules of the binary regression model. The sample size was calculated as follows:

$$n\approx \frac{3\mathrm{EPV}*6}{\pi }.$$

### 2.5 Statistical analyses

Mean and standard deviation ($\bar{x}$± s) or median and interquartile ranges [M (IQR)] were applied to describe quantitative data. Categorical variables are shown as number (*n*) or rate (%). The distribution of all continuous data was tested using the Shapiro–Wilk (SW) test. Group comparisons of quantitative data were performed by the independent sample *t*‐test or Mann–Whitney U test, and differences in qualitative data in both groups were compared by the chi‐square test or Fisher test. *p* < .05 indicated the statistical significance. The multicollinearity of all related variables was explored by tolerance (Tol) and variance inflation factor (VIF). Tol > 0.1 or VIF<10 suggested almost noncollinearity. The parameters with *p* < .05 by univariate regression analysis were further subjected to stepwise forward multivariate logistic regression. The effects of related variables are presented as the odds ratio (OR) with 95% confidence interval (CI) and the *p* values. Besides, the Hosmer and Lemeshow goodness‐of‐fit test was implemented to examine the model fitness of the logistic regression.

The TDI was calculated by the regression coefficients of related variables from the multivariate model, which was further conducted with univariate logistic regression analysis to ascertain the correlation with POD. TDI = 0.122 × age + 0.463 × average VAS scores within the postoperative first 3 days−0.045 × PWR. Moreover, the accuracy of TDI and its component factors in predicting POD was described by the receiver operating characteristic curve (ROC) analysis. IBM SPSS statistics software version 25.0 (SPSS Inc., Chicago, Illinois, USA) was performed to analyze all data.

## 3 RESULTS

### 3.1 Comparison of demographic characteristics and perioperative data

**FIGURE 1:**
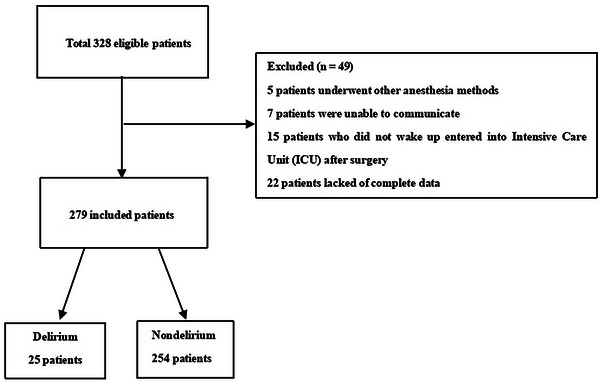
Flow chart of the study population.

During this study period, a total of 328 patients undergoing thoracic surgery were enrolled. After removing some patients according to the exclusion criteria, the remaining 279 cases were ultimately analyzed, of whom 25 cases were defined as POD (Figure 1). The number of enrolled patients met the requirements of the binary regression model.

As shown in Table 1, the POD incidence was approximately 8.96%, and the overall median (interquartile ranges) age was 67 (64–71). Among demographic data, patients with delirium were older than those without delirium (70 (67–74) versus 67(63–71), *p* = .011). In addition, blood infusion (*p* = .030) and average VAS scores in the first 3 days after surgery (*p* = .023) had significant differences between the two groups.

**TABLE 1:** Demographic and perioperative data of all patients.

Variables	Overall (*N* = 279)	Delirium (*n* = 25)	Nondelirium (*n* = 254)	*p*‐value
Age (years)	67 (64–71)	70 (67–74)	67 (63–71)	.011^*^
Gender (male)	119 (42.7)	9 (36)	110 (43.3)	.481
BMI (kg/m^2^)	25.1 (22.5–27.4)	25.2 (22.3–26.5)	25.1 (22.5–27.5)	.471
ASA grade, *n* (%)				
I	1 (0.4)	0 (0)	1 (0.4)	.196
II	136 (48.7)	8 (32)	128 (50.4)	
III	142 (50.9)	17 (68)	125 (49.2)	
Educational level, *n* (%)				
Low degree (illiteracy and primary school)	103 (36.9)	9 (36)	94 (37)	.434
Medium degree (middle and senior school)	161 (57.7)	16 (64)	145 (57.1)	
High degree (college and above)	15 (5.4)	0 (0)	15 (5.9)	
CCI	3 (3–4)	4 (3–5)	3 (3–4)	.058
Smokers, *n* (%)	51 (18.3)	7 (28)	44 (17.3)	.188
Alcohol abuse, *n* (%)	23 (8.2)	2 (8)	21 (8.3)	.963
Cognitive impairment, *n* (%)	23 (8.2)	3 (12)	20 (7.9)	.738
Anxiety, *n* (%)	33 (11.8)	5 (20)	28 (11)	.185
Depression, *n* (%)	7 (2.5)	1 (4)	6 (2.4)	.617
Hypertension, *n* (%)	132 (47.3)	14 (56)	118 (46.5)	.362
Cardiac arrhythmia, *n* (%)	33 (11.8)	3 (12)	30 (11.8)	.978
Coronary heart disease, *n* (%)	36 (12.9)	2 (8)	34 (13.4)	.650
Usage of antiarrhythmics, *n* (%)	21 (7.5)	3 (12)	18 (7.1)	.623
Usage of steroids, *n* (%)	24 (8.6)	0 (0)	24 (9.4)	.145
Usage of benzodiazepines, *n* (%)	13 (4.7)	3 (12)	10 (3.9)	.184
Duration of anesthesia (min)	190 (140–260)	245 (140–327.5)	190 (140–250)	.106
Duration of surgery (min)	85 (140–192)	180 (92.5–265)	137.5 (85–190)	.086
Surgical methods(endoscopic), *n* (%)	267 (95.7)	24 (96)	243 (95.7)	.938
Types of diseases, *n* (%)				
Lung cancer	227 (81.4)	19 (76)	208 (81.9)	.210
Esophageal tumor	24 (8.6)	2 (8)	22 (8.7)	
Tracheal tumor	3 (1.1)	1 (4)	2 (0.8)	
Hiatal hernia	6 (2.2)	2 (8)	4 (1.6)	
Mediastinal tumor	15 (5.4)	1 (4)	14 (5.5)	
Chest wall tumor	4 (1.4)	0 (0)	4 (1.6)	
Dosage of opioids				
Remifentanil (mg)	1.3 (0.9–2.1)	1.9 (1–3.3)	1.3 (0.9–2)	.080
Sufentanil (μg)	25 (20–30)	25 (25–30)	2 5(20–30)	.150
Vasoactive drugs usage, *n* (%)	59 (21.1)	7 (28)	52 (20.5)	.379
Estimated blood loss volume (mL)	131.0 (54.0–242.0)	154 (76.5–266)	128.5 (52–236.8)	.447
Urine volume (mL)	300 (200–500)	300 (150–1250)	300 (187.5–500)	.182
Blood transfusion, *n* (%)	11 (3.9)	3 (12)	8 (3.1)	.030^*^
Minimum body temperature (°C)	36.3 (36.1–36.4)	36.3 (36.2–36.5)	36.3 (36.1–36.4)	.174
Use of postoperative analgesic pump, *n* (%)	232 (83.2)	20 (80)	212 (83.5)	.659
Average VAS scores within the postoperative first 3 days	4.3 (3.3–5)	5 (3.8–6)	4.3 (3.3–5)	.023^*^
Total times of analgesics used within the postoperative first 3 days	1 (0–1)	1 (0–1.5)	1 (0–1)	.214
Post‐surgical stay (days)	5 (4–7)	6 (4–9.5)	5 (4–7)	.114
Length of hospital stay (days)	9 (7–13)	12 (7–17)	9 (7–13)	.100

Abbreviations: ASA, American Society of Anaesthesiologists; BMI, body mass index;  CCI, Charlson comorbidity index; VAS, Visual Analogue Scale.

* *p*<.05.

Regarding laboratory indicators, as demonstrated in Table 2, the increased levels of white blood cells, neutrophils, monocytes, creatinine, and d‐dimer were observed in the POD group (all *p*<.05). In contrast, The levels of PWR, hemoglobin, albumin, GNRI, calcium, and sodium were lower in the POD group compared with that in the non‐POD group (all *p*<.05).

**TABLE 2:** Preoperative laboratory indicators between two groups.

Variables	Overall (*N* = 279)	Delirium (*n* = 25)	Nondelirium (*n* = 254)	*p*‐value
White blood cell count (×10**^9^**/L)	6.6 (5.2–9.1)	8.4 (6.3–12)	6.5 (5.1–9)	.008^*^
Neutrophil count (×10**^9^**/L)	4.6 (3.3–7.3)	6.8 (3.7–10.2)	4.4 (3.2–7)	.025^*^
Total lymphocyte count (×10**^9^**/L)	1.4 (1–1.9)	1.4 (0.8–2.4)	1.4 (1–1.8)	.683
Monocyte count (×10**^9^**/L)	0.3 (0.3–0.5)	0.4 (0.3–0.6)	0.3 (0.3–0.5)	.024^*^
Platelets count (×10**^9^**/L)	221 (182–256)	195 (153.5–241)	222.5 (188–258)	.054
Neutrophil‐to‐lymphocyte ratio	2.9 (1.8–7.6)	4.2 (1.7–12.3)	2.9 (1.8–7.4)	.369
Platelet‐to‐lymphocyte ratio	159.8 (121.1–213.2)	137.6 (93.3–246.6)	162 (123.2–211.9)	.150
Platelet‐to‐WBC ratio	33.6 (22.2–44.2)	23.2 (15.6–33.7)	34.4 (22.8–44.7)	.002^*^
Monocyte‐to‐lymphocyte ratio	0.2 (0.2–0.4)	0.4 (0.2–0.7)	0.2 (0.2–0.4)	.060
Hemoglobin (g/L)	128.6 ± 15.0	121.6 ± 15.8	129.3 ± 14.8	.015^*^
Serum albumin (g/L)	39.2 (36–42.4)	36.9 (33.3–39.8)	39.5 (36.2–42.4)	.017^*^
GNRI	105.4 (98.9–112.7)	99 (96–106.3)	105.8 (99.2–113.1)	.014^*^
Calcium (mmol/L)	2.3 (2.1–2.4)	2.1 (2–2.3)	2.3 (2.1–2.4)	.026^*^
Sodium (mmol/L)	140 (138–142)	139 (135.5–141)	140 (139–142)	.026^*^
AST/ALT	1.3 (1–1.5)	1.4 (1.1–1.6)	1.3 (1–1.5)	.404
Creatinine (μmol/L)	65.1 (55.9–76.3)	71.2 (60.2–82.7)	64.5 (55.1–75.7)	.031^*^
LDH (U/L)	176.5 (159.1–198.9)	186.5 (160.7–202.6)	175.9 (159–199.1)	.649
d‐dimer (mg/L)	0.4 (0.3–0.7)	0.7 (0.4–1.3)	0.4 (0.3–0.6)	.001^*^

Abbreviations: AST/ALT, aspartate transaminase/alanine; GNRI, geriatric nutritional risk index;

LDH, lactate dehydrogenase; WBC, white blood cell.

* *p* < .05.

### 3.2 Multicollinearity analysis among potential variables

The results presented in Supplementary Table 1 display severe collinearity between white blood cells and neutrophil count. Considering the correlation between the PWR and white blood cell count, there was no collinearity among the variables after eliminating the white blood cell count (all Tol>0.1, VIF<10), as presented in Supplementary Table 2.

### 3.3 Related risk factors for POD and the establishment of TDI

In the beginning, all potential factors with *p*<.05 were put into univariate regression analysis, and our results demonstrated unadjusted factors including age, average VAS scores within the postoperative first 3 days, hemoglobin, serum albumin, GNRI, calcium, sodium, PWR, white blood cell, and neutrophil count had a statistical significance in both groups. Furthermore, the independent risk factors for POD using multivariate regression analysis were age (OR: 1.130; 95% CI: 1.041–1.227; *p* = .003), PWR (OR: 0.956; 95% CI: 0.922–0.991; *p* = .015), and average VAS scores within the postoperative first 3 days (OR: 1.589; 95% CI: 1.122–2.249; *p* = .009), as indicated in Table 3.

**TABLE 3:** Univariate and multivariate logistic regression analyses of potential variables for POD.

Variables	Univariate					Multivariate				
	B	Wald	OR	95% CI	*p*‐value	B	Wald	OR	95% CI	*p*‐value
Age (years)	0.104	7.093	1.110	1.028–1.198	.008^*^	0.122	8.559	1.130	1.041–1.227	.003^*^
Red blood cells (U)	0.315	2.166	1.370	0.901–2.084	.141	–	–	–	–	–
Plasma (ml)	0.003	3.580	1.003	1.000–1.006	.058	–	–	–	–	–
Average VAS scores within the postoperative first 3 days	0.458	7.353	1.582	1.136–2.203	.007^*^	0.463	6.815	1.589	1.122–2.249	.009^*^
White blood cell count (×10^9^/L)	0.123	4.966	1.131	1.015–1.260	.026^*^	–	–	–	–	–
Neutrophil count (×10^9^/L)	0.116	4.572	1.123	1.010–1.249	.033^*^	–	–	–	–	–
Monocyte count (×10^9^/L)	0.983	2.170	2.672	0.723–9.875	.141	–	–	–	–	–
Platelet‐to‐WBC Ratio	‐0.050	7.904	0.951	0.919–0.985	.005^*^	‐0.045	5.881	0.956	0.922–0.991	.015^*^
Hemoglobin (g/L)	‐0.033	5.708	0.968	0.942–0.994	.017^*^	–	–	–	–	–
Serum albumin (g/L)	‐0.104	5.840	0.902	0.829–0.981	.016^*^	–	–	–	–	–
GNRI	‐0.044	4.437	0.957	0.918–0.997	.035^*^	–	–	–	–	–
Calcium (mmol/L)	‐3.717	7.461	0.024	0.002–0.350	.006^*^	–	–	–	–	–
Sodium (mmol/L)	‐0.204	7.950	0.861	0.708–0.940	.005^*^	‐0.135	3.165	0.874	0.753–1.014	.075
Creatinine (μmol/L)	0.018	3.644	1.018	1.000–1.036	.056	–	–	–	–	–
d‐dimer (mg/L)	0.177	2.706	1.193	0.967–1.473	.100	–	–	–	–	–

*Note*: Hosmer and Lemeshow goodness‐of‐fit test: χ2 value = 3.227, *p* = .919. Abbreviations: CI, confidence interval; GNRI, geriatric nutritional risk index; OR, odds ratio; VAS, Visual Analogue Scale; WBC, white blood cell.

* *p* < .05.

**FIGURE 2:**
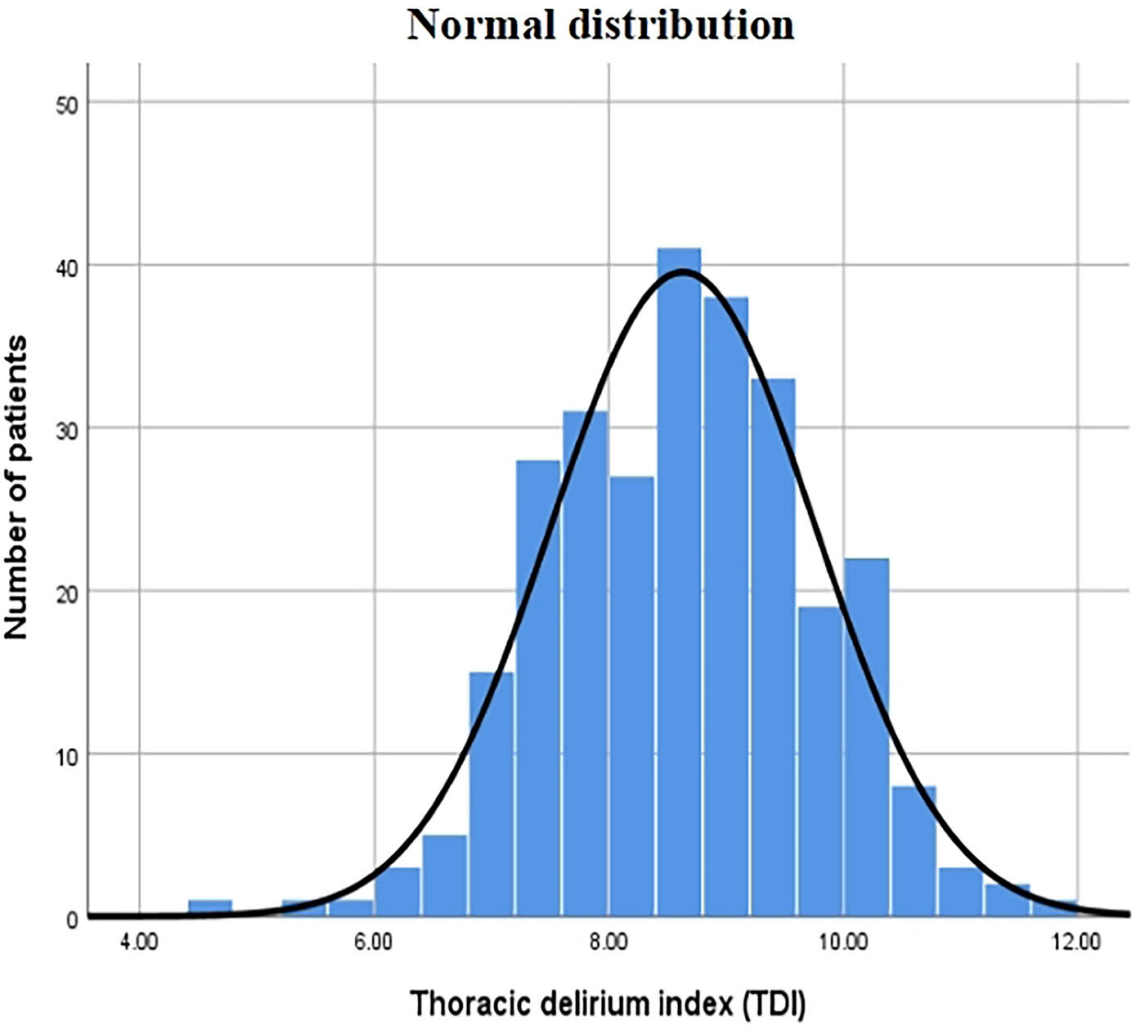
Distribution of TDI.

Moreover, TDI was established by the regression coefficients of the determined risk factors. Supplementary Table 3 reveals the significant difference in terms of TDI between the two groups (*p* < .001). The distribution of TDI conformed to the normality by the SW test (*p* = .494) (Figure 2). Besides, our results manifested the correlation between TDI and POD by the univariate regression analysis (Supplementary Table 4).

### 3.4 The accuracy of TDI and its component factors for POD by ROC analysis

**FIGURE 3:**
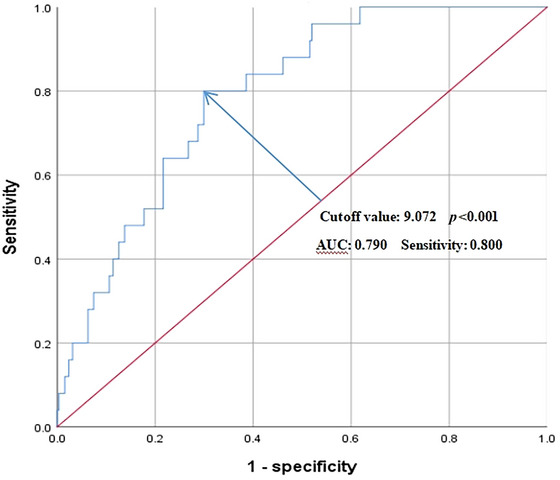
The predictive value of TDI for POD by ROC analysis.

Figure 3 displays the ROC curve of TDI, and the results in Table 4 show that the area under the curve (AUC) of 0.790 was the largest in TDI, accompanied by the optimal cutoff value of 9.072, in comparison with its component factors, which suggested TDI had a superior predictive value for POD.

**TABLE 4:** The accuracy of TDI and its component factors by ROC curve analyses.

Variables	Area under the curve (95% confidence interval)	Sensitivity	Specificity	Cutoff value	*p*‐value
Age (years)	0.653 (0.539–0.768)	0.680	0.370	68.5	.011
Average VAS scores within the postoperative first 3 days	0.637 (0.515‐0.759)	0.360	0.098	5.5	.024
Platelet‐to‐WBC Ratio	0.684 (0.573–0.796)	0.496	0.200	34.455	.002
TDI	0.790 (0.715–0.866)	0.800	0.299	9.072	<.001

Abbreviations; GNRI, geriatric nutritional risk index; TDI, thoracic delirium index; VAS, Visual Analogue Scale; WBC, white blood cell.

## 4 DISCUSSION

This study clarified that the independent risk factors for POD after thoracic surgery were age, PWR, and average VAS scores within the first 3 days after surgery. Meanwhile, TDI was weighed and established based on these factors, which may be a more useful tool for predicting POD following thoracic surgery with its simplicity and practicality.

Similar to some prior studies (Hayashi et al., 2019; Ozyurtkan et al., 2010), the prevalence of POD was 8.96% in this study. Nevertheless, other studies have reported a lower incidence of POD following thoracic surgery, such as 5.32% and 3.3% (Ishibashi et al., 2022; Yildizeli et al., 2005), which may be due to the differences in included age range, sample size, diagnostic criteria, and surgical types. Currently, few studies investigated risk factors for POD in thoracic surgery. A prior study found that age, current smoking status, depression, and peripheral vascular disease were positively associated with POD after primary lung cancer surgery (Hayashi et al., 2019). Another study suggested that advanced age, longer surgical time, and abnormal postoperative chemical markers (e.g., potassium, sodium, and glucose) would increase the likelihood of POD in thoracic surgery (Yildizeli et al., 2005). Similarly, age was also correlated with the occurrence of POD in our study. Notably, PWR and average VAS scores within the postoperative first 3 days were recognized as independent risk factors for POD in the present study, while not recognized in some previous studies. Therefore, larger multicenter studies should be needed to further assess risk factors for POD after thoracic surgery in the future.

It is widely considered that older patients increase the risk of developing POD (Méndez‐Martínez et al., 2021). Despite the pathogenesis remains obscure that may be explained by the fact that age‐induced microglial priming results in hypersensitivity to stimuli (Bugiani, 2021). European Delirium Association and American Delirium Society illuminate that delirium is a sign of brain vulnerability and aging can lead to a decline in the brain's functional reserve, thus elderly patients may be more likely to have delirium (American Delirium Society 2014). Moreover, more comorbidities and complex medication use in geriatric patients may increase the likelihood of POD (Kassie et al., 2019; Ramos et al., 2022). Our results also further proved that older patients were more prone to develop delirium after surgery. So, surgeons and anesthesiologists should pay more attention to neurological damage in elderly patients following thoracic surgery.

Additionally, there were significant statistical differences in PWR, white blood cell, and neutrophil counts in the two groups. Several studies noted that neuroinflammation might have a crucial effect on the progression of POD, and neutrophils and lymphocytes were the major effectors of neuroinflammation among elderly patients with delirium, manifested as increased neutrophil count and decreased lymphocyte count (Kotfis et al., 2019; Oh & Park, 2019). As studies mentioned, a high level of NLR has been identified as an independent risk factor for POD in acute ischemic stroke and orthopedic surgery (Guldolf et al., 2021; Li et al., 2022). Also, other inflammatory markers ratios, such as MLR and PLR, played a prominent role in predicting POD after cardiac surgery (Şaşkin et al., 2022; Su et al., 2022). Interestingly, a recent study showed the connection between higher PWR and poor clinical consequences in patients with acute ischemic stroke (Amalia & Dalimonthe, 2020). In our study, PWR has also verified its correlation with POD after adjusting associated risk factors by multivariate regression analysis. To our knowledge, our study first demonstrated PWR as a meaningful predictor for POD following thoracic surgery, and the lower level of PWR was relevant to a higher risk of POD, which further provided important evidence for the underlying relationship between dysfunction of immune effector cells and delirium. Taken together, the abnormal level of some derived white blood cell ratios (NLR or PWR) might depict the degree of subclinical inflammation, which could lead to an increased risk of neurological disorders in the elderly. Similar to our outcomes, a retrospective study revealed that PWR was recognized as a valuable predictor for POD following abdominal surgery (Ida et al., 2020). Another study by Kotfis et al. (2019) also further evidenced that the lower level of PWR might be involved in the episode of POD in patients undergoing cardiac surgery. These studies clearly suggested that the changes in systemic inflammation factors before the surgical stimulus, namely increased leukocyte, decreased lymphocyte count, and thrombocytopenia, might be beneficial to predicting the risk of POD.

POD often occurred within the first 3 days postoperatively, of which neuroinflammation was one of the common physiological mechanisms (Xiao et al., 2023). Surgical trauma could lead to the release of inflammatory mediators (e.g., TNF‐α and IL‐1, etc.) and ultimately produce postoperative pain, which was often correlated with anxiety, depression, sleep disturbances, and postoperative cognitive dysfunction (Matsuda et al., 2019). One study by Gold et al. (2022) showed that acute pain was a high‐risk factor for POD in elderly patients after orthopedic surgery. Hence, early reduction of patients' postoperative pain would prevent POD to some extent, which might be related to lower levels of inflammatory factors. Thoracic surgery is well‐known as one of the most painful surgical procedures for open or minimally invasive surgery, and postoperative pain often causes restlessness and other uneasiness in patients (Thompson et al., 2018). Notably, postoperative pain in the first 3 days after surgery could promote the occurrence of POD to a certain degree in the present study, which was consistent with prior studies (Gold et al., 2022; Ma et al., 2022). Specifically, higher VAS scores could dramatically add to the risk of the onset of POD (OR: 1.589; 95% CI: 1.122–2.249; *p* = .009), which suggests an early and appropriate postoperative analgesia management program might effectively reduce the incidence of POD, and thus facilitating the postoperative recovery of geriatric patients undergoing thoracic surgery. Unfortunately, the postoperative analgesic remedy had no statistical differences in both groups, which might have something to do with the refusal of some patients to analgesic usage.

In addition, we established a novel TDI based on screened independent risk factors by multivariate regression analysis. Our results noted TDI had the largest AUC of 0.790 and the highest sensitivity of 0.800 in comparison with other factors, indicating that TDI might be a more practical predictor of POD. Many researchers have developed their own predictive indicators of POD in other types of surgery. Wang et al. (2021) performed a retrospective study to develop a risk stratification index model (RIS) by identifying risk factors for POD in the elderly with hip fractures, and their results showed that the POD incidence could significantly increase when RIS ≥ 5. Moreover, another study investigated the connection between some preoperative serum biomarkers and POD following abdominal surgery and established PLR and PWR index to better predict POD (Ida et al., 2020). Hence, establishing TDI might exert a promoting effect on reducing POD in older people following thoracic surgery.

Several limitations need to be addressed. First, delirium was only evaluated in the postoperative first 3 days, which may underestimate POD incidence. Second, our study merely established a predictive index. Nonetheless, a stratified analysis of risk factors may better predict POD. Third, some other risk factors for POD, such as sleep disorder, hypotension, the duration of one‐lung ventilation, and so on, were not included in this study. Fourth, we did not collect and compare the types of thoracic surgery due to the lack of relevant data.

## 5 CONCLUSION

In summary, we identified independent risk factors for POD after thoracic surgery and developed TDI to better prevent POD. The predictive factors were three well‐defined clinical variables in our study, including age, PWR, and average VAS scores within the first 3 days after surgery, which were easy to measure and calculate. Meanwhile, TDI could early predict the risk of POD in older patients after thoracic surgery. Clinicians should consider the TDI as a part of evidence‐based prevention strategies to identify geriatric patients at risk of POD following thoracic surgery.

## AUTHOR CONTRIBUTIONS

**Jianli Li**: Funding acquisition; project administration; writing—review and editing. **Jing Liu**: Conceptualization; data curation; writing—original draft; writing—review and editing. **Mingming Zhang**: Formal analysis; software. **Jing Wang**: Investigation. **Meinv Liu**: Methodology. **Dongdong Yu**: Methodology. **Junfang Rong**: Software; supervision.

## CONFLICT OF INTEREST STATEMENT

All authors declared no conflict of interest existed in the manuscript.

### PEER REVIEW

The peer review history for this article is available at https://publons.com/publon/10.1002/brb3.3379.

## Supporting information


**Supplementary Table 1** Collinearity analysis of all related variables

**Supplementary Table 2** Collinearity analysis of other variables after excluding white blood cell

**Supplementary Table 3** TDI in elderly patients with or without delirium

**Supplementary Table 4** Result of univariate logistic regression analysis in TDI

## Data Availability

All data that support the findings of this study are available upon request from the corresponding author.
