# Supplementary material for: Thoracic delirium index for predicting postoperative delirium in elderly patients following thoracic surgery: A retrospective case‐control study
Source: Brain Behav. 2024 Jan 8;14(1):e3379. doi: 10.1002/brb3.3379 (PMC10772846; doi:10.1002/brb3.3379)
Supplement: Supplementary file 1 — Supplementary Table 1 Collinearity analysis of all related variables [file BRB3-14-e3379-s003.docx]

**Supplementary Table 1** **Collinearity analysis of all related variables**

| Collinear statistics | | |
| --- | --- | --- |
| Variables | Tol | VIF |
| Age (years) | 0.944 | 1.060 |
| Average VAS scores  within the postoperative first 3 days | 0.973 | 1.027 |
| White blood cell count (×109/L) | 0.044 | 22.484 |
| Neutrophil count (×109/L) | 0.044 | 22.951 |
| Platelet-to-WBC Ratio | 0.478 | 2.090 |
| Hemoglobin (g/L) | 0.751 | 1.331 |
| Serum albumin (g/L) | 0.352 | 2.843 |
| GNRI | 0.837 | 1.195 |
| Calcium (mmol/L) | 0.381 | 2.624 |
| Sodium (mmol/L) | 0.886 | 1.129 |
| GNRI: Geriatric Nutritional Risk Index Tol:Tolerance VAS:Visual Analogue Scale  VIF:Variance inflation factor WBC: White Blood Cell | | |
