# Supplementary material for: Thoracic delirium index for predicting postoperative delirium in elderly patients following thoracic surgery: A retrospective case‐control study
Source: Brain Behav. 2024 Jan 8;14(1):e3379. doi: 10.1002/brb3.3379 (PMC10772846; doi:10.1002/brb3.3379)
Supplement: Supplementary file 2 — Supplementary Table 2 Collinearity analysis of other variables after excluding white blood cell [file BRB3-14-e3379-s002.docx]

**Supplementary Table 2** **Collinearity analysis of other variables after excluding white blood cell**

| Collinear statistics | | |
| --- | --- | --- |
| Variables | Tol | VIF |
| Age (years) | 0.947 | 1.056 |
| Average VAS scores  within the postoperative first 3 days | 0.975 | 1.026 |
| Neutrophil count (×109/L) | 0.463 | 2.160 |
| Platelet-to-WBC Ratio | 0.482 | 2.074 |
| Hemoglobin (g/L) | 0.777 | 1.286 |
| Serum albumin (g/L) | 0.353 | 2.834 |
| GNRI | 0.837 | 1.194 |
| Calcium (mmol/L) | 0.385 | 2.598 |
| Sodium (mmol/L) | 0.897 | 1.115 |
| GNRI: Geriatric Nutritional Risk Index Tol:Tolerance VAS:Visual Analogue Scale  VIF:Variance inflation factor WBC: White Blood Cell | | |
