# Supplementary material for: Thoracic delirium index for predicting postoperative delirium in elderly patients following thoracic surgery: A retrospective case‐control study
Source: Brain Behav. 2024 Jan 8;14(1):e3379. doi: 10.1002/brb3.3379 (PMC10772846; doi:10.1002/brb3.3379)
Supplement: Supplementary file 3 — Supplementary Table 3 TDI in elderly patients with or without delirium [file BRB3-14-e3379-s004.docx]

**Supplementary Table 3 TDI in elderly patients with or without delirium**

| **Variables** | **Overall**  **(N =279)** | **Delirium**  **(n =25)** | **Non-delirium**  **(n =254)** | ***P*-value** |
| --- | --- | --- | --- | --- |
| TDI | 8.6±1.1 | 9.6±0.9 | 8.5±1.1 | ＜0.001* |
| TDI: Thoracic Delirium Index * *P*＜0.05 | | | | |
