# Supplementary material for: Thoracic delirium index for predicting postoperative delirium in elderly patients following thoracic surgery: A retrospective case‐control study
Source: Brain Behav. 2024 Jan 8;14(1):e3379. doi: 10.1002/brb3.3379 (PMC10772846; doi:10.1002/brb3.3379)
Supplement: Supplementary file 4 — Supplementary Table 4 Result of univariate logistic regression analysis in TDI [file BRB3-14-e3379-s001.docx]

**Supplementary Table 4 Result of univariate logistic regression analysis in TDI**

| **Variables** | **Univariate** | | | | |
| --- | --- | --- | --- | --- | --- |
|  | **B** | **Wald** | **OR** | **95% CI** | ***P*-value** |
| TDI | 1.073 | 20.439 | 2.926 | 1.837-4.659 | ＜0.001* |
| CI: Confidence Interval OR: Odds Ratio * *P*＜0.05 TDI: Thoracic Delirium Index | | | | | |
